# Pre-ablation and Post-ablation Factors Influencing the Prognosis of Patients with Electrical Storm Treated by Radiofrequency Catheter Ablation: An Update

**DOI:** 10.31083/j.rcm2512432

**Published:** 2024-12-05

**Authors:** Cosmin Cojocaru, Maria Dorobanțu, Radu Vătășescu

**Affiliations:** ^1^Department of Cardiothoracic Pathology, Faculty of Medicine, “Carol Davila” University of Medicine and Pharmacy, 050474 Bucharest, Romania; ^2^Department of Cardiology, Emergency Clinical Hospital of Bucharest, 014461 Bucharest, Romania; ^3^Romanian Academy, 010071 Bucharest, Romania

**Keywords:** electrical storm, catheter ablation, risk stratification

## Abstract

Catheter ablation-based management strategies for the drug-refractory electrical storm (ES) have been proven to abolish acute ventricular arrhythmic episodes and improve long-term outcomes. However, this effect is highly influenced by multiple independently acting factors, which, if identified and addressed, may allow a more tailored management to each particular case to improve results. This review synthesizes existing evidence concerning ES outcome predictors of patients undergoing ablation and introduces the role of novel scoring algorithms to refine risk stratification. The presence of these factors should be assessed during two distinct phases in relation to the ablation procedure: before (based on preprocedural multimodal evaluation of the patient’s structural heart disease and comorbidities) and after the ablation procedure (in terms of information derived from the invasive substrate characterization, procedural results, postprocedural recurrences (spontaneous or during non-invasive testing), and complications).

## 1. Introduction and Brief Review of Terminology

**Fig. 1.**
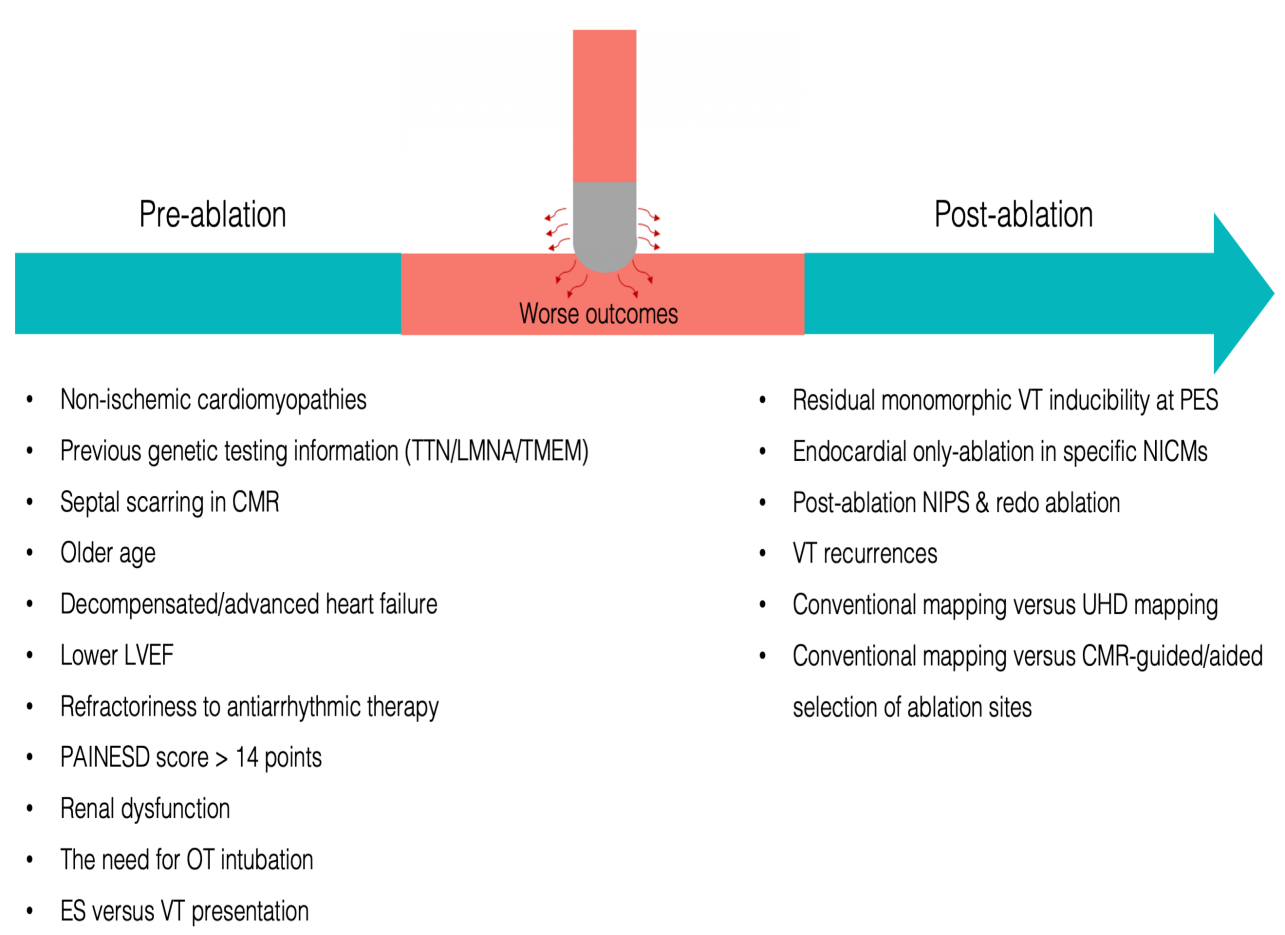
**Risk factors that can be identified pre- and post-ablation to negatively influence the prognosis after catheter ablation for an electrical storm**. PES, programmed electrical stimulation; LVEF, left ventricular ejection fraction; RFCA, radiofrequency catheter ablation; CS, cardiogenic shock; CMR, cardiac magnetic resonance; UHD mapping, ultra-high-density mapping; TTN, titin; VT, ventricular tachycardia; OT, orotracheal; ES, electrical storm; LMNA, lamin A/C; TMEM, transmembrane protein; NICMs, non-ischemic cardiomyopathies; NIPS, non-invasive programmed ventricular stimulation.

Catheter ablation-based management strategies for drug-refractory electrical storm (ES) can acutely suppress ventricular arrhythmic episodes and may improve long-term outcomes [1]. Mortality and recurrences of ES patients are strongly dependent on the presence of structural heart disease, ablation results, procedural complications, and non-cardiovascular comorbidities (Fig. 1) [2, 3, 4, 5]. Thus, it is pivotal to identify parameters with evidence-based impact on outcomes after ablation for ES, which may enable tailored treatment. Novel scoring algorithms with improved accuracy for event prediction have recently emerged and can be implemented in clinical practice [6, 7, 8, 9, 10].

This review synthesizes existing evidence concerning ES outcome predictors of patients undergoing ablation and introduces the role of novel scoring algorithms to refine risk stratification. The presence of these factors should be assessed during two distinct phases in relation to the ablation procedure: before (based on preprocedural multimodal evaluation of the patient’s structural heart disease and comorbidities) and after the ablation procedure (in terms of information derived from the invasive substrate characterization, procedural results, postprocedural recurrences (spontaneous or during non-invasive testing), and complications).

The current terminology distinguishes between isolated episodes of ventricular tachycardia (VT) and incessant or recurrent VTs that cluster within a short interval because there are significantly more severe outcomes in the latter scenario compared to the former [11, 12, 13, 14, 15]. Although arbitrarily and variably defined in previous publications, an ES is defined in the most recent European consensus document as at least three distinct (separated by at least 5 minutes of baseline rhythm) episodes of ventricular arrhythmia (either monomorphic/polymorphic VT and/or ventricular fibrillation (VF)) requiring anti-arrhythmic intervention and clustered in a 24-hour interval or by the presence of incessant VT [1, 11, 16, 17, 18, 19, 20]. Incessant VT is defined by immediate (under 5 minutes) VT recurrences despite successful termination by anti-tachycardia pacing or electrical shock and persisting for over 12 hours [21]. Although this definition mainly refers to internal cardioverter defibrillator (ICD) recipients, it may also be used for those without cardiac implantable electronic devices (CIEDs) experiencing repeated and sustained VT [18, 22]. It is, however, important to emphasize that recent data show that even two episodes of ventricular arrhythmia appearing within a 3-month interval are associated with clear incremental mortality [23]. This is why a “one-size-fits-all” definition of an ES based on a specific number of VT episodes and a certain time interval may not be sufficient; hence, immediate evaluation and assessment of treatment options for any episode of VT is needed. Data refining ES epidemiology, baseline characteristics, and optimal care strategies are still expanding (i.e., the ELECTRA registry [24]). Regarding the type of VT, ES predominantly manifests through episodes of sustained monomorphic VT (SMVT), defined by a constant beat-to-beat QRS morphology [18, 25, 26, 27]. Polymorphic VT (PVT) is characterized by continuously changing QRS morphology due to fast modifications in the ventricular activation sequence [18]. Anti-arrhythmic drug (AAD)-refractory ES (which mandates the cardiologist refer the patient to catheter ablation promptly) is defined by most recent European guidelines [13, 28] as the lack of response to beta-blockers, sedation, and intravenous amiodarone administration. Moreover, it is clinically intuitive that AAD-refractory ES requires catheter ablation after correction of precipitating or reversible factors and ICD optimization (to avoid adequate yet unnecessary therapies for self-limiting arrhythmia) [11, 13, 16].

It is important to understand that the role and methods of radiofrequency catheter ablation (RFCA) procedures can vary in patients experiencing an ES. The most common RFCA indication addresses substrate in drug-refractory scar-related SMVT in patients with structural heart disease (SHD) [13], irrespective of the ischemic (ICM) or non-ischemic (NICM) substrate (class IB indication). However, RFCA may also target specific arrhythmic triggers, such as premature ventricular contractions leading to PVT in various scenarios, including primary electrical diseases (class IIa indication) [13]. In summary, the main mapping principles are represented by substrate mapping and ablation (targeting late potentials (LPs), local abnormal ventricular activities (LAVAs), scar homogenization or scar dechanneling), activation mapping, pace mapping, and entrainment mapping [29].

Defining ablation outcomes is essential to properly characterize the procedure’s effect on the substrate (and ultimately to anticipate the long-term clinical course), yet debates are still underway among experts regarding the “perfect result”. Indeed, this is demonstrated by the recent publication by Santangeli *et al*. [30] concerning optimal ablation endpoints (this study revisited a ten-year-old paper published by the same authors [31]). In this sense, VT non-inducibility at end-procedural PES is consistently the most validated “positive” result of ablation. In contrast, complete elimination of LAVA has been linked to superior survival and less recurrent VT, although this may not always be achieved in “real-life” clinical practice [30, 32, 33, 34].

Most studies concerning RFCA in an ES have considered the response to end-procedural programmed electrical stimulation (PES) protocols as a surrogate for procedural success or failure [14, 35, 36, 37, 38]. However, concordance between end-procedural and non-invasive CIED-based follow-up PES (non-invasive programmed ventricular stimulation, (NIPS)) in the search for residual VT has been demonstrated to be suboptimal and, more especially, prognostic (this will be addressed later during this review) [39]. Hence, an appropriate endpoint for RFCA is considered to achieve non-inducibility of any SMVT or at least of the “clinical” preprocedural morphology, whereas the latter is due to persistent inducibility of any monomorphic VT not previously clinically documented [39, 40, 41, 42]. Very fast SMVT (cycle lengths <200–250 ms) or PVT/VF are usually not considered to be prognostic and are therefore not generally considered a sign of procedural failure (or at least associated with a considerably lower future risk of events [35, 43, 44]). Finally, up to 10% of patients may not tolerate or consent to end-procedural PES; thus, the procedural result is usually labeled as “not tested” [14].

Table 1 (Ref. [14, 35, 37, 38, 42, 45, 46, 47, 48, 49, 50, 51, 52, 53, 54, 55, 56, 57, 58, 59]) summarizes the results of existing studies concerning the results of RFCA in an ES.

**Table 1.** **Results for ES and ventricular tachycardia catheter ablation from existing studies or meta-analyses/systematic reviews**.

Authors	Number of ES patients treated by RFCA	Etiology	Monomorphic VT non-inducibility	PES protocol	Endo-epicardial approach
Nayyar *et al*. [45]	471 (meta-analysis)	ICM 68%	Eliminated all SMVT in 72%, clinical SMVT non-inducible 91%	NR (non-homogenous)	NR
		NICM 32%			
Izquierdo *et al*. [46]	23	ICM 82.6%	PES only in 16/23 patients—9 with SMVT non-inducibility	NR (non-homogenous)	NR
		NICM 17.4%			
Koźluk *et al*. [47]	24	ICM 75%	NR	NR	Epicardial ablation in 8.3%
		NICM 25%			
Özcan *et al*. [48]	44	ICM	Eliminated all SMVT in 55.5%, clinical SMVT non-inducible in 90.8%	NR	Only endocardial
Carbucicchio *et al*. [38]	95	ICM 75%	Eliminated all SMVT in 72%, clinical SMVT non-inducible in 89%	Up to 3 ESx	Epicardial ablation in 11.1%
		NICM 25%			
Vergara *et al*. [14]	677	ICM 54.7%	Eliminated all SMVT in 63.9%, clinical SMVT non-inducible in 93%	Up to 3 ESx, not tested in 6.5%	Epicardial ablation in 27.1%
		NICM 45.3%			
Muser *et al*. [35]	267	ICM 74.5%	Eliminated all SMVT in 73%	Up to 3 ESx, not tested in 10%	Epicardial ablation in 18%
		NICM 26.5%			
Kumar *et al*. [49]	287	ICM 64.8%	Eliminated all SMVT in 60% ICM/50% NICM, clinical SMVT non-inducible in 89% ICM/82% NICM	Up to 3 ESx, not tested in 9% ICM/18% NICM	Epicardial ablation in 3.8% ICM/24% NICM
		NICM 35.2%			
Vătășescu *et al*. [42]	82	ICM 68.2%	Eliminated all SMVT in 69.5%, clinical SMVT non-inducible in 92.6%	Up to 4 ESx	Epicardial ablation in 11.3% ICM, 48.1% NICM
		NICM 31.8%			
Jin *et al*. [50]	54	ICM	Eliminated all SMVT in 80%, clinical SMVT non-inducible in 95%	Up to 3 ESx	Only endocardial
Mueller *et al*. [51]	108	ICM 45%	Eliminated all SMVT in 76%, clinical SMVT non-inducible in 94%	Up to 4 ESx	Epicardial ablation in 27%
		NICM 55%			
Laredo *et al*. [52]	23	NICM (AC)	Eliminated all SMVT in 46%, clinical SMVT non-inducible in 92%	NR	Epicardial ablation in 17%
Huang *et al*. [53]	58	ICM 38%	Eliminated all SMVT in 53%, clinical SMVT non-inducible in 79%	Up to 4 ESx, not tested in 5%	Epicardial ablation in 5%
		NICM 62%			
Kozeluhova *et al*. [54]	50	ICM 76%	Eliminated all SMVT in 40%, clinical SMVT non-inducible in 84%	Up to 3 ESx, not tested in 8%	Epicardial ablation in 8%
		NICM 24%			
Di Biase *et al*. [37]	92	ICM	Eliminated all SMVT in 100%	Up to 3 ESx	Epicardial ablation in 33% of the endo-epicardial homogenization subgroup
Mueller *et al*. [55]	107	ICM 45%	Eliminated all SMVT in 63% in the presence of septal substrate, 87% in the absence of septal substrate	Up to 3 ESx	NA
		NICM 55%			
Deneke *et al*. [56]	32	ICM 53.1%	Eliminated all SMVT in 59.3%, clinical SMVT non-inducible 93.7%	Up to 3 ESx	Epicardial ablation in 9.3%
		NICM 46.9%			
Arya *et al*. [57]	13	NICM (DCM)	Eliminated all SMVT in 61.5%	Up to 3 ESx	Epicardial ablation in 30.7%
Ballout *et al*. [58]	21	ICM 81%	Eliminated clinical VT in 71% (included PVC-induced VT/VF)	Up to 3 ESx, not tested in 19%	Epicardial ablation in 9.5%
		NICM 19% (cardiogenic shock)			
***Žižek *et al*. [59]	60 primary prevention ICD recipients	ICM 100% (primary prevention substrate modification by ablation)	Reduced post-ablation rate of ES and appropriate ICD therapies	Up to 3 ESx	NA

ES, electrical storm; ICM, ischemic cardiomyopathy; NICM, non-ischemic cardiomyopathy; SMVT, sustained monomorphic ventricular tachycardia; PES, programmed electrical stimulation; VT, ventricular tachycardia; NR, not reported; ESx, extra stimuli; AC, arrhythmogenic cardiomyopathy; DCM, dilated cardiomyopathy; NA, not available; PVC, premature ventricular contraction; VF, ventricular fibrillation; ICD, internal cardioverter defibrillator; ***, primary prevention ablation in ICD recipients with infarct-related chronic total coronary occlusions; RFCA, radiofrequency catheter ablation.

## 2. Factors to be Considered before Performing Ablation for Electrical Storm

Firstly, catheter ablation results are worse in ES settings compared to VT patients without ES criteria in terms of end-procedural residual VT inducibility (71.2% *vs*. 63.9%), and PES is more frequently avoided (usually due to the higher risk of hemodynamic deterioration) [14]. Furthermore, contemporary larger-scale cohorts have shown that ES itself is independently associated with a three-fold higher risk for early postprocedural mortality [60].

Etiology (ICM versus NICM) was initially expected to influence end-procedural residual VT inducibility significantly. The HELP-VT study was among the first to show that, using modern RFCA strategies, VT ablation can lead to non-inducibility in up to approximately 73% of both ICM and NICM patients [61], subsequently confirmed by other high-volume centers [35]. However, the analysis of a more numerous cohort by Kumar *et al*. [62] suggested that end-procedural PES is more likely to induce residual non-clinical VT in NICM subgroups. Overall, meta-analysis-derived data indicate that acute procedural results may ultimately be worse in NICM regarding inducibility, yet this should be further evaluated when larger samples are available for inclusion [63]. Nonetheless, NICM procedural results imply more complexity regarding the need for epicardial ablation (up to 30–54% versus 1–5%) and longer procedural times [35, 61]. Additionally, evidence regarding VT ablation in NICM suggests significant heterogeneity in results as the lowest rates of non-inducibility (44%) are achieved in valvular cardiomyopathies, whereas PES can become negative after ablation in up to 80% of arrhythmogenic cardiomyopathies [64]. In patients with ICM, the presence of chronic total coronary occlusions increases the rate of residual VT inducibility at end-procedural PES after ablation [65]. Moreover, the rate of major complications seems similar in ICM compared to NICM VT ablations [66]. There are different complication profiles in relation to etiology: ICM is more frequently affected by vascular-access-related problems (2-fold) and cerebrovascular events (8-fold), whereas NICM patients required more surgical drainage for pericardial effusions (which is to be expected considering 45% of NICM required endo-epicardial ablation compared to 8% in the ICM subgroup) [66]. Interestingly, Ding *et al*. [66] observed the lack of changes in the rate of VT ablation complications over time [66], which may result from the interaction between more advanced and safe ablation strategies and techniques with more severe contemporary patient profiles referred for ablation. Etiology does not seem to increase mortality after ES ablation (although there is a trend toward higher mortality in ICM patients) [67]. The risk of VT recurrences in NICM is increased by 53% compared to ICM cohorts [14]. However, this has been challenged by more recent large-scale cohorts showing similar recurrence rates (46%) in both ICM and NICM patients over a median follow-up of 45 months [35].

Genetic testing can aid the preprocedural multimodal evaluation of the substrate [68]. Mutations in lamin A/C (*LMNA*) and titin (*TTN*) genes most frequently create substrates in anteroseptal basal segments, whereas those with mutations affecting phospholamban (*PLN*), sodium voltage-gated channel alpha subunit 5 (*SCN5A*), desmoplakin (*DSP*), and ribonucleic acid binding motif protein 20 (*RBM20*) genes show a predominant inferolateral involvement [69]. Septal scarring significantly increases the rate of SMVT inducibility at PES (due to the greater difficulty in targeting the substrate adequately) [55]. Although based on a relatively small cohort, VT ablation in *TTN* mutations most frequently targeted periaortic and basal septal regions, resulting in 20% residual inducibility of other SMVT morphologies [70]. Half of the patients required extensive ablation from both sides of the interventricular septum due to intramural circuits [70]. Transmembrane protein (*TMEM*) mutations leading to arrhythmogenic cardiomyopathy more frequently display biventricular substrate and more inducible VT than non-*TMEM* genotypes [71]. *LMNA* mutations induce deep intramural septal scarring requiring multiple ablations targeting the basal anterior septum, which often causes/aggravates conduction disorders [72]. Up to 40% of patients with dilated cardiomyopathies referred for a RFCA test were positive for pathogenic or likely pathogenic variants (P/LPv) [69, 73]. The presence of P/LPv independently doubles the risk of VT recurrence during follow-up and significantly increases the rate of mortality and need for cardiac transplantation (HTx) [69]. In *TTN* mutations, although acute procedural results appear to be in line with previous general NICM studies, recurrences are very frequent (53% with multiple ablations, 33% after first ablation), mostly during the first 3 months post-ablation, whereas mortality reached 27% during a median interval of 26.5 months [70].

With regard to these results, although stemming from VT ablation studies, acute heart failure (HF) severity seems not to affect acute procedural results when end-procedural testing remains possible [74]. However, PES is not possible for up to 11% of patients in the New York Heart Association (NYHA) IV class, which is significantly more frequent than in the NYHA II–III classes (5%) [74]. In the most extreme setting (i.e., cardiogenic shock requiring mechanical circulatory support due to refractory arrhythmia), bail-out RFCA was proven efficient, and although mortality is high (29%), it allows device-weaning for a substantial proportion of patients [58, 75].

Considering its simplicity for evaluating systolic performance, left ventricular ejection fraction (LVEF) has recurrently been described as a predictor of death and recurrences. Each unit of LVEF reduction induces up to a 4.9% higher risk of death and a 2.3% higher risk of recurrences [14, 35, 76, 77]. HF severity is also an important driver of mortality after ES ablation [14]. NYHA IV class patients have higher in-hospital and long-term mortality (17% in-hospital and 48% at 1 year) and present with early VT recurrences more frequently (up to 19% of cases) compared to those with NYHA II/III class HF (possibly due to more advanced SHD) [74]. Notably, early VT recurrences (<1 month post-ablation) induce an 8-fold higher risk of death in NYHA IV class patients [74].

Amiodarone treatment has been proven to influence both acute and long-term RFCA results. Meanwhile, the substrate seems to display higher complexity (in terms of late potential and local abnormal ventricular activity) in patients not receiving amiodarone treatment during RFCA for VT [78]. Furthermore, “off” amiodarone patients require epicardial ablation more frequently to achieve non-inducibility and longer procedures with longer radiofrequency applications [78]. Furthermore, in terms of follow-up NIPS results, patients “off” amiodarone have significantly less chances of VT inducibility after ablation [79]. This may be explained by isthmuses being “hidden” by the anti-arrhythmic effects during ablation, which may later become apparent, especially after AAD discontinuation. Hence, if possible, withholding amiodarone for multiple days before ablation and re-testing for VT inducibility by NIPS after a certain interval of post-ablation amiodarone interruption may uncover residual substrate [79]. Similarly, although acute results seem superior in those undergoing VT ablation while under general anesthesia compared to mild sedation (either as a “true positive” effect by less respiratory movements affecting catheter stability or as a confounding factor due to the potential antiarrhythmic impact of anesthesia), the risk of subsequent VT recurrence due to incomplete substrate characterization is still being questioned [80, 81].

Performing preprocedural imaging (cardiac magnetic resonance (CMR) or cardiac-computed tomography substrate characterization) may aid the electrophysiologist in anticipating the location of relevant arrhythmogenic isthmuses. This may guide VT ablation and improve acute results through better substrate characterization [82, 83]. Selection of ablation sites guided by CMR significantly enhanced the rate of non-inducibility (82%) compared to CMR-aided (68%) and no-CMR-aided (54%) strategies in shorter procedural times and with similar rates of complications [82, 84, 85].

Ablation timing is highly important for adequate management and to prevent complications. A relatively recent multicenter study [5] showed that the timing of RFCA strongly influences short-term 30-day mortality and emphasizes that an initial timeframe for heart failure stabilization of >48 hours in ES patients before proceeding to catheter ablation can improve outcomes (if the arrhythmic episodes can be transiently suppressed by medication). This mortality benefit was even higher if the delayed (>48 hours) procedure did not induce VT [5]. However, this strategy should not lead to further unnecessary delay in performing VT ablation, as Huang *et al*. [53] have shown that RFCA during the index procedure reduces VT recurrence, ES recurrence, and re-hospitalization compared to medical therapy as an initial strategy and a longer history of recurrent multiple ICD therapies appears also to aggravate outcomes [86]. Hence, this analysis suggests that up-titration of AADs should not be preferred *in lieu* of RFCA for patients presenting with ES, especially because acute recurrence rates are very high [87].

The preprocedural presence of certain factors should raise attention to a higher risk of periprocedural complications. Major complications are regarded as events leading to long-term disability, requiring intervention (including transfusions), or prolonged hospitalization [88, 89]. Certain high-risk operators, such as creatinine levels >1.3 mg/dL and age >70 years old, were shown to independently predict complications in a multivariate analysis by Peichl *et al*. [88] concerning VT ablation. Age influences the safety of VT ablation procedures as major complications are significantly more frequent in those older than 80 years (up to 18.5% of cases), with lower procedural success rates [90]. Older age is also associated with longer hospitalization [90]. Previous reports show renal dysfunction has independently predicted mortality after ES ablation [91]. In addition, short-term mortality (throughout the first 30 days after ES initiation) is significantly driven by pre-hospital cardiac arrest and the need for orotracheal intubation [92]. The rate of major complications during ES ablation does not seem higher compared to VT ablation [14, 88] and varies between 2% and 7% (most commonly represented by vascular access complications such as pseudoaneurysms and pericardial effusions leading to tamponade) [14, 35, 45, 66, 89].

## 3. Factors to be Considered during or after Performing Ablation for Electrical Storm

The end-procedural testing protocol employed at PES significantly influences the acute results. Most representative studies based PES protocols on two or three extra stimuli (ESx) [14, 35]. It has been shown that more aggressive four ESx stimulations may uncover up to 17% more patients that have inducible VT who were otherwise labeled as non-inducible with three ESx based; meanwhile, up to 30% of all patients with residual inducibility are diagnosed using four ESx protocols [93, 94]. Importantly, this seems to translate into more frequent clinical post-ablation events [93, 94]. This means that even if aggressive testing can reduce the “complete non-inducibility” rate, it can reveal more residual arrhythmogenic substrates. Notably, Santangeli *et al*. [31] have previously emphasized that VT induction during PES is probabilistic rather than deterministic. Hence, aside from more aggressive stimulation protocols, targeted sites during PES may influence inducibility as stimulation within the scar doubles the chance of inducing clinical VT and reduces the risk of only non-clinical VT induction compared to standard RV pacing [95].

Dual endo-epicardial approach, in comparison to the endocardial approach, seems not to impact residual VT inducibility, except for certain specific etiologies such as arrhythmogenic cardiomyopathies or chronic myocarditis, which are expected to have more extensive epicardial than the endocardial substrate [37, 96, 97]. However, the endo-epicardial approach may reduce long-term VT recurrences [37, 96, 97]. In addition, data derived from meta-analyses [98, 99] advocate for implementing remote-magnetic navigation systems as they provide two-fold higher acute success in VT ablation. Identification and ablation of the endocardial and epicardial relevant substrate by endo-epicardial ablation in ICM have been shown to reduce the probability of VT recurrence by 62% compared to endocardial ablation alone [37]. Interestingly, this improved outcome was present despite an identical (100%) rate of SMVT non-inducibility at the end of RFCA for both endocardial-only and endo-epicardial subgroups. The authors considered that even if endocardial ablation may transiently modify the acute electrical properties of intramural or epicardial segments, rendering the re-entry circuits non-functional, they may become active once again during long-term follow-up, leading to VT recurrences [37]. The presence of septal substrate in an ES increases the risk of death (surprisingly, with no effect on recurrences) during follow-up [55].

Acute ablation results are among the strongest predictors of both death/transplantation and VT recurrences during follow-up [14, 35, 42, 46, 57]. Obtaining non-inducibility for SMVT with cycle lengths >250 ms at PES reduces the risk of death and recurrences by over 75% in long-term follow-ups [14, 35]. Data from the most numerous ES cohort demonstrated that 86.3% of patients with complete non-inducibility were alive one year after the ES ablation. In contrast, those with RFCA failure (by residual inducibility for clinical VT) have a significantly lower survival rate (51.2%) [14]. Importantly, in certain models, non-inducibility does not remain an independent mortality predictor after adjusting for VT recurrence during follow-up [35]. This suggests that the effect of residually inducible VT on death is mediated by arrhythmic reoccurrence. Further, persistent VT inducibility was the only independent predictor (with a 5-fold higher probability) of long-term VT recurrences in one of the largest ES ablation cohorts currently published [35]. It seems that postprocedural NIPS and the decision to perform redo ablation prior to hospital discharge in patients with residually inducible VT during the first days after an index ablation can improve long-term outcomes by increasing the number of patients achieving non-inducibility [67, 100]. This is highly relevant as NIPS becomes a prognostic tool and an adequate indicator of the need for reintervention (especially after RFCA lesions stabilize, the sympathetic periprocedural drive reduces, and better hemodynamic status is obtained) [67, 100]. Furthermore, patients who obtain non-inducibility for any SMVT have better survival than those not tested (who are frequently frail or have developed acute HF) [14, 74].

Predictably, VT recurrences during follow-up can be regarded as a central factor for worse outcomes as they induce a 5-fold higher mortality/need for cardiac transplantation in multivariable models [35]. Vergara *et al*. [14] showed that one-year survival in ES patients with recurrences was 61.3%, whereas 88.7% of those without recurrences were alive after one-year post-RFCA.

Ultra-high density (UHD) three-dimensional scar mapping provides a deeper understanding of scar functional anatomy. Recent data showed that although acute procedural results are similar, RFCA guided by UHD three-dimensional scar mapping based on a 64-electrode mapping basket catheter independently predicted better survival and lower recurrences over a 21.1-month interval [101]. Moreover, this was not at the cost of more radiation exposure or periprocedural complications [101]. CMR-guided ablations have translated into lower rates of VT recurrences compared to VT ablations performed with no CMR-derived data [82]. Furthermore, although not reaching statistical significance, VT ablations guided by computed tomography-based dedicated protocols (“In-HEART” software) of substrate visualization were more successful in terms of recurrences in both ICM and NICM compared to standard RFCA strategies [102].

The use of intracardiac echocardiography (ICE) during VT ablation reduces radiation exposure for high-volume centers, improves intraprocedural visualization of anatomical landmarks, and has been shown to independently reduce the rate of VT-recurrence-related readmissions and the need for redo ablations [103]. However, visual characterization of certain substrate types is suboptimal by ICE (particularly in epicardial mapping and left ventricular summit arrhythmia, where fluoroscopy is essential for adequately identifying coronary arteries and electrophysiology catheters and guidewires) [104]. Optimization of ablation strategies by imaging is currently expanding. The VOYAGE multicenter randomized trial is currently recruiting patients for VT ablation to assess the impact of CMR-guided ablations on long-term recurrences [105].

The need for redo ablations has also been studied after VT ablation (although not specifically in ES settings) [106, 107]. Firstly, the profile of patients requiring redo VT ablations is significantly different in terms of presentation (more frequently ES), etiology (more frequently NICM), history of cardiac surgery and receiving more AADs, have more periprocedural complications and higher long-term mortality [106]. This is why ablation results are worse (41.8% of patients are still inducible after repeat ablations compared to 32.9% after first ablation) [106]. Notably, if redo ablation is successful in preventing recurrent VT episodes, patients obtain similar mortality compared to those not requiring redo procedures. In summary, even if redo ablations are more difficult, obtaining long-term control of VT episodes leads to better survival. A recent study published by Garcia Garcia *et al*. [107] showed that redo ablations can reduce the burden of shocks and VT by 73% during the first year; almost one in four patients die (predominantly due to terminal HF) or require HTx or circulatory support, and 46% have recurrences. Factors aggravating prognosis were advanced NYHA class, anteroseptal compared to the inferolateral substrate, and procedural complications [107].

The effect of withholding amiodarone long-term treatment in patients with end-procedural RFCA non-inducibility has also been questioned. Liang *et al*. [79] demonstrated that amiodarone reduction or discontinuation after obtaining RFCA non-inducibility is possible with no incremental VT recurrence rates. Considering the potential systemic adverse effects of amiodarone, the authors propose this practice as a goal after procedural success [79].

## 4. Scoring Algorithms to be Considered for Risk Stratification in ES Patients Treated by Radiofrequency Catheter Ablation

**Fig. 2.**
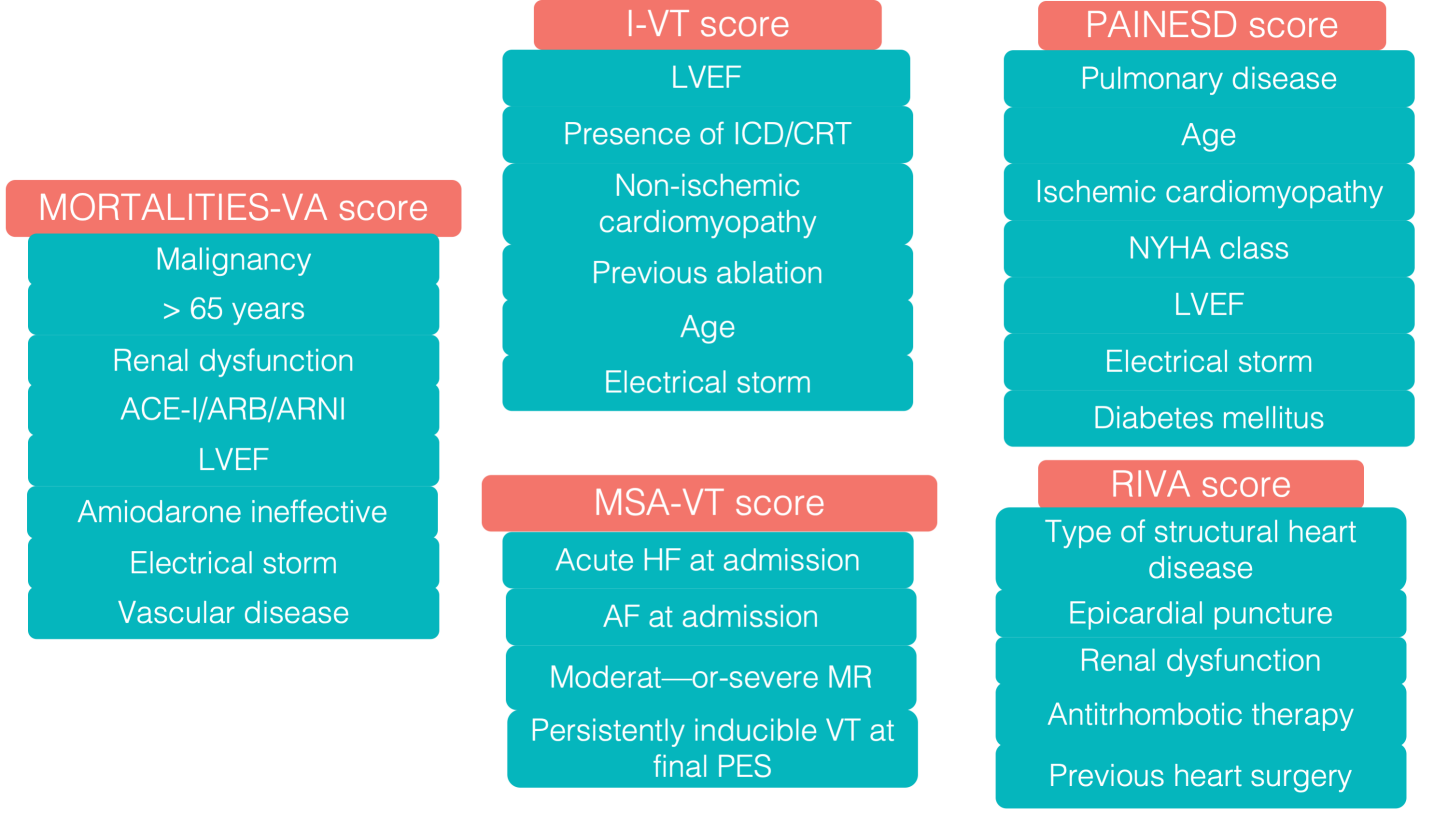
**Scoring algorithms for risk stratification in ventricular tachycardia ablation**. NYHA, New York Heart Association; ICD, internal cardioverter defibrillator; ACE-I, angiotensin-converting enzyme inhibitor; ARB, angiotensin receptor blocker; ARNI, angiotensin receptor blocker and neprylisin inhibitor; LVEF, left ventricular ejection fraction; VT, ventricular tachycardia; PES, programmed electrical stimulation; MR, mitral regurgitation; HF, heart failure; AF, atrial fibrillation; CRT, cardiac resynchronization therapy.

Mathew *et al*. [89] developed the RIVA score (“risk in VT ablation”) (Fig. 2), which predicts a combined endpoint represented by major periprocedural complications and in-hospital death of patients treated by RFCA for VT. This score accounts for multiple factors associated with a specific number of points based on logistic regression analysis to estimate the risk of in-hospital events. Thus, structural heart disease increases risk (especially in the presence of idiopathic cardiomyopathies), the need for epicardial access, renal dysfunction, ongoing antithrombotic treatment, and the lack of cardiac surgery history (although the authors’ interpretation of this last factor is not clarified) [89]. More recently, a modified version of the score entitled “mRIVA” was proposed by Doldi *et al*. [108], which added NYHA class and age as adjunctive factors that appear to improve the prediction of in-hospital events after VT ablation.

The PAINESD algorithm was created to identify patients at risk for hemodynamic decompensation and acute HF during VT ablation, which could benefit from periprocedural mechanical circulatory support (pre-emptive extracorporeal membrane oxygenation (ECMO)) [8]. This score is based on the presence of multiple characteristics: pulmonary disease (5 points), age >60 years (3 points), ICM (6 points), NYHA III/IV classes (6 points), LVEF <25% (3 points), ES (5 points), and diabetes mellitus (3 points). High-risk patients are defined by scores higher than 14 points, and almost one in four are expected to develop periprocedural acute HF [8]. Importantly, one in two patients who experience periprocedural acute HF die during the index hospitalization [8]. The I-VT SCORE algorithm was among the first to derive and validate a decisional model that estimates long-term risks of death and VT recurrence [9], which can be applied both pre-procedurally and after performing end-procedural PES to include RFCA results. LVEF was the most informative parameter for recurrence and death prediction, with an optimal cut-off threshold of 30%. Furthermore, etiology (ICM *vs*. NICM), presence of a CIED, and repeated ablations influenced recurrences, whereas ES presentation, previous ablations, very severely depressed (LVEF <14%), and old age (>80 years) affected mortality [9]. In addition, the authors confirmed that the previously published PAINESD score [6, 7, 8] can predict long-term outcomes; however, it is less accurate compared to the I-VT score (with or without PES result inclusion).

The MORTALITIES-VA scoring algorithm appears to accurately predict death or the need for cardiac transplantation after VT ablation [10]. This score relies on multiple comorbidities to identify the risk of recurrent events: malignancy (4 points), older age >65 years (3 points), renal dysfunction (5 points), LVEF <35% (6 points), ineffective amiodarone (4 points), ES presentation (3 points), and vascular disease (4 points), whereas treatment with angiotensin-conversion enzyme inhibitors, angiotensin receptor blockers and angiotensin receptor blockers–neprylisin inhibitors had a protective effect (–3 points). Patients with more than 15 points had a 70% risk of death or need for cardiac transplantation during follow-up.

The MSA-VT scoring algorithm showed the role of moderate-or-severe mitral regurgitation, atrial fibrillation at admission, acute heart failure symptoms at admission, and persistently inducible sustained monomorphic VT at end-procedural PES after ES ablation [109]. An MSA-VT score ≥3 points correlated with significantly higher mortality and VT recurrences after ES ablation.

Recently, machine-learning-based scoring systems have attempted to improve risk stratification through multiparametric calculations after VT ablation and have proven to have superior prediction efficiency compared to PAINESD and I-VT scores [110]. The most important predictors of recurrence in the published model were several VT morphologies, ES presentation, LVEF and LV dilation, and the severity of mitral regurgitation. In this area, the role of artificial intelligence imaging processing is expanding in certain centers to aid in non-invasive scar characterization, prediction of possible VT isthmuses, and selection of optimal ablation sites [111, 112, 113]. However, these are still under development and have not yet been validated in clinical designs.

## 5. Potential Future Therapies to Improve Outcomes for Patients with Electrical Storm

Ultra-low-temperature cryoablation (ULTC) using –196 °C N_2_ cryogen in VT ablation has recently been reported as feasible and safe for patients with scar-dependent VT in both ischemic and non-ischemic cardiomyopathies (Cryocure-VT trial) [114]. This first-in-human multicenter experience showed acute VT non-inducibility for 94% of patients and 81% 6-month freedom of ICD shocks. Similarly, the role of stereotactic arrhythmia radio-ablation (STAR) for patients with unsuccessful or contraindications for catheter ablation has recently been explored in multiple small-scale study groups and has shown a promising reduction in VT burden during follow-up [115, 116, 117, 118, 119], specifically in ES settings [120]. Furthermore, initial experience with pulsed-field ablation technology for VT suppression by electroporation of myocyte membranes has been reported and seems favorable [121, 122, 123]. However, long-term follow-up data on these novel therapeutic approaches is still lacking, and research efforts are underway.

The role of substrate-modification by ablation for primary prevention of ventricular arrhythmia is emerging and has been demonstrated by the recent PREVENTIVE-VT multicenter trial, which assessed the effect on outcomes of ablation versus withholding ablation in primary prevention ICD recipients with infarct-related chronic total occlusions (CTOs) [59]. Importantly, “preventive ablation” significantly reduced the occurrence of ES episodes, the rate of appropriate ICD therapies, and also reduced cardiac hospitalizations. However, it did not impact mortality rates in this population.

In contrast to myocardial lesion-based therapies, the role of regenerative techniques is currently being explored in experimental settings. Human embryonic stem cell-derived cardiomyocytes (hESC-CMs) have shown the potential to reduce susceptibility to spontaneous and inducible VT episodes in injured areas of myocardium transplanted with hESC-CMs grafts by host-graft electromechanical integration phenomenon [124]. Furthermore, experimental models with epicardially-sutured biocompatible materials such as carbon nanotube fibers (CNTfs) have been reported, seeking to improve conductibility across fibrosis-affected myocardium to avoid forming local re-entry circuits [125]. Surgical peri-scar deployment of a fibroblast-infused biomaterial has also been shown to improve local ventricular refractory intervals and has reduced (although without reaching statistical significance) the rate of inducible VT in a recent murine-model-based experience [126].

## 6. Conclusions

In conclusion, catheter ablation is a pivotal treatment of drug-refractory electrical storm in patients with structural heart disease. If non-inducibility is obtained at end-procedural testing, long-term outcomes regarding mortality and recurrences improve. However, multiple other specific factors have been proven to foreshadow subsequent major adverse cardiovascular events. Therefore, identifying such predictors is important before, during, and after the ablation procedure. Furthermore, new risk stratification algorithms accounting for multiple, simultaneously acting parameters are emerging and may be actively implemented in clinical practice.
